# Mechanical and Physicochemical Properties of 3D-Printed Agave Fibers/Poly(lactic) Acid Biocomposites

**DOI:** 10.3390/ma14113111

**Published:** 2021-06-05

**Authors:** Valeria Figueroa-Velarde, Tania Diaz-Vidal, Erick Omar Cisneros-López, Jorge Ramón Robledo-Ortiz, Edgar J. López-Naranjo, Pedro Ortega-Gudiño, Luis Carlos Rosales-Rivera

**Affiliations:** University Center of Exact Sciences and Engineering (CUCEI), University of Guadalajara, Guadalajara 44430, Mexico; valeria.fvelarde@alumnos.udg.mx (V.F.-V.); taniadzv@gmail.com (T.D.-V.); erick.cisneros@academicos.udg.mx (E.O.C.-L.); jorge.robledo@academicos.udg.mx (J.R.R.-O.); edgar.lopezn@academicos.udg.mx (E.J.L.-N.); pedro.ogudino@academicos.udg.mx (P.O.-G.)

**Keywords:** agave fibers, 3D printing, fused deposition modeling, poly(lactic) acid, biocomposites

## Abstract

In order to provide a second economic life to agave fibers, an important waste material from the production of tequila, filaments based on polylactic acid (PLA) were filled with agave fibers (0, 3, 5, 10 wt%), and further utilized to produce biocomposites by fused deposition modeling (FDM)-based 3D printing at two raster angles (−45°/45° and 0°/90°). Differential scanning calorimetry, water uptake, density variation, morphology, and composting of the biocomposites were studied. The mechanical properties of the biocomposites (tensile, flexural, and Charpy impact properties) were determined following ASTM international norms. The addition of agave fibers to the filaments increased the crystallinity value from 23.7 to 44.1%. However, the fibers generated porous structures with a higher content of open cells and lower apparent densities than neat PLA pieces. The printing angle had a low significant effect on flexural and tensile properties, but directly affected the morphology of the printed biocomposites, positively influenced the impact strength, and slightly improved the absorption values for biocomposites printed at −45°/45°. Overall, increasing the concentrations of agave fibers had a detrimental effect on the mechanical properties of the biocomposites. The disintegration of the biocomposites under simulated composting conditions was slowed 1.6-fold with the addition of agave fibers, compared to neat PLA.

## 1. Introduction

Among the available rapid prototyping or additive manufacturing technologies, printing based on fused deposition modeling (FDM) using thermoplastics is the best for modeling possibilities, mainly due to its simplicity, versatility, low economic impact, and widespread applicability [1]. FDM, also known as 3D printing, allows three-dimensional figures to be obtained at high speed and precision, based on the extrusion of a polymer filament in a layer-by-layer arrangement (X and Y axes) on top of a movable plate (*z* axis), until a 3D structure is obtained [2]. These complex figures can be produced in less time, as the prior formation of a mold is not required [3]. FDM applications have seen a predominant growth in personal prototyping fabrication, mainly due to a sharp cost reduction of FDM machines [4]. However, commercial FDM applications are still leading the market, mainly for the formation of automotive parts and aerospace prototypes [5], electronic devices, house construction, among others [6,7]. Promising results have also been found in the field of medicine for the production of biomedical devices and materials, such as bone replacement scaffolds [8,9,10].

Common polymer matrices employed in FDM techniques include acrylonitrile butadiene styrene (ABS), polycarbonates (PC), polyamides, polystyrene (PS), polymethyl methacrylate (PMMA), polyethylene (PE), and polylactic acid (PLA) [11]. Recently, efforts have been focused on the production and use of sustainable and renewable bioplastics (such as PLA) for a wide variety of applications, from packaging to engineering, and biomedical devices, replacing petroleum-based plastics [12,13,14]. PLA is a thermoplastic biopolymer belonging to the family of aliphatic polyesters. PLA is obtained by fermentation of renewable resources such as corn or agricultural waste [15]. Unlike other polymers, PLA is considered a sustainable material as it can be compostable, which can be further utilized to promote a new cycle of agricultural products for future conversion into PLA [16].

The innate versatility of rapid prototyping enables the fabrication of high-resolution parts reinforced with diverse materials such as ceramics, metals, minerals, and natural fibers [6,17]. Traditional reinforcing materials such as glass or carbon fibers allow the fabrication of composites with superior mechanical properties; however, their inherent inconvenience relies on the difficulty of recycling their components once the material is no longer purposeful [6]. In this sense, the addition of natural fibers as reinforcers in composite materials has gained great attention due to their “green” character, cheaper cost, and high availability [6,18]. The production of biocomposites from renewable sources, recycled fibers, industrial co-products, and forestry and agricultural residues is an environmentally friendly approach for the production of materials with a lower carbon footprint, reducing the ecological impact of waste materials [6]. Fabrication of 3D-printed PLA-based biocomposites using natural fibers includes bamboo powder [19], wood flour [20], wood [21], kraft lignin [22], and cork [23], among others [24].

In 2020, the Tequila Regulatory Council estimated the production of 523,600 tons of bagasse from *Agave tequilana* Weber var. Azul. Thus, 1.4 kg of agave waste is created per liter of produced tequila [25,26], posing an extreme environmental issue. Several researchers have joined efforts to find low-cost, environmentally friendly alternative uses to agave wastes. Agave residues can be treated to obtain fibers that are typically short and elliptically shaped, 10–12 cm long, and with a diameter of 592.34 μm [27]. Agave fibers have been previously used for the obtaining of biocomposites. Torres-Tello produced biocomposites from agave fibers and polyhydroxybutyrate (PHB) with increased tensile and flexural moduli [28]. Recently, PLA- and agave fiber-reinforced biocomposites were produced by dry-blending and rotational molding, demonstrating the production of biodegradable, low-cost biocomposites [29]. However, to date, there have been no published reports concerning reinforcement with agave fibers to produce filaments for an FDM process for a final application in 3D printing. As agave fibers are waste material, the addition of waste fibers to matrices, which are usually expensive, can help to diminish the cost of the material. This offers the opportunity to generate novel materials suitable for 3D printing with certain advantages, such as lightness, sustainability, biodegradability, and low cost (compared to neat polymeric materials).

The present work describes the development of a biodegradable filled material for further use as filaments in a 3D printer. Agave fibers (mesh 100–140) were used as a filler material in a PLA matrix. The modified variables were weight percentage (0, 3, 5, and 10 wt%) and FDM deposition angle (−45°/45° and 0°/90°). Differential scanning calorimetry (DSC), water uptake, density variation, and biodegradability of the biocomposites were studied, whereas morphological characterization was performed via SEM. The mechanical properties were studied following the ASTM international norms for tensile, flexural, and Charpy impact. The effects of FDM process parameters on the tensile properties of fabricated agave fiber/PLA biocomposites were also investigated.

## 2. Materials and Methods

### 2.1. Materials

Polylactic acid 3251D (PLA, density = 1.24 g cm^−3^, melt flow index = 80 g/10 min^−1^, (2.16 kg, 210 °C), according to the provided technical data sheet), supplied in spherical pellets, was obtained from Nature Works LLC (Minnetonka, MN, USA). Agave fibers (AF) from *A. tequilana* Weber var. Azul were obtained from a local tequila company (Mundo Agave, Tequila, Mexico).

### 2.2. Agave Fiber Cleaning and Sieving

AF were prepared as reported by Pérez-Fonseca et al. [30] with minor modifications. Briefly, the pithy fibers were hydrated for 24 h, and next, the pith was separated from the fiber with a two-disk (diameter, 30 cm) Sprout-Waldron D2A509NH refiner (Muncey, PA, USA). The separated AF were then centrifuged for 20 min at 3750× *g* rpm to remove excess water, and dried outdoors. Finally, the AF were ground with a ball mill and sieved using a Ro-Tap RX-29 (Oelde, Germany) with a #100–140 sieve fraction.

### 2.3. AF/PLA Filament Preparation

Samples of AF with different weight percentages (0, 3, 5, 10 wt%) and PLA were prepared using a total weight of 1000 g for each composition (Table S1, Supplementary Materials). Next, the materials were dry-blended using a high shear mixer JR Torrey LP-12 (Monterrey, Mexico) for 2 min and oven-dried at 60 °C for 24 h. The filaments were then produced by extrusion using a twin-screw extruder Leistritz Micro 27 GL/GG 32D (Nuremberg, Germany) with a screw rotational speed of 65 rpm. A forward ramp temperature profile with nine zones was set to 160/160/165/165/170/170/175/175/175 °C. The final filament had a diameter of 1.7 ± 0.07 mm.

### 2.4. Differential Scanning Calorimetry (DSC)

A differential scanning calorimeter (TA Instruments Q100 (New Castle, DE, USA)) was used to study the thermal properties of the agave-fibers/PLA filaments. The specimens were cut to a homogenous weight of 3–5 mg and heated once from 25 to 200 °C (10 °C min^−1^) under nitrogen atmosphere. Glass transition temperature $\left(T_{g}\right)$, cold crystallization temperature $\left(T_{\mathrm{cc}}\right)$, melting temperature $\left(T_{m}\right)$, enthalpy of cold crystallization $\left({\Delta H}_{\mathrm{cc}}\right),$ and melting enthalpy $\left({\Delta H}_{m}\right)$ were determined from the DSC curves with TRIOS TA software (Version 4.4.0.41651) [31]. Values of $T_{m}$ and ${\Delta H}_{m}$ were obtained from the peak temperature and area of the melting endotherm, respectively.

The crystallinity level $\left(X_{c}\right)$ of the samples was evaluated from their corresponding melting enthalpies using Equation (1) [32],

(1) $$X_{c}\left(\%\right)=\frac{{\Delta H}_{m}-{\Delta H}_{\mathrm{cc}}\;}{\Delta H{}^{\circ}}\times \frac{100}{w}$$

where ΔH° (PLA) is the melting enthalpy (93.7 J g^−1^) of 100% crystalline (perfect crystal) PLA, and w is the weight fraction of PLA in the biocomposites.

### 2.5. Fused Deposition Modeling/3D Printing

Computer aided designs (CAD) were drawn and visualized in Google SketchUp (Version 16.0.14004, Mountain View, CA, USA) [33] with dimensions following the ASTM standards for each test/characterization. The designs were transferred to a printable format using the Hot-World GmbH & Co. Repetier-Host software [34] (Version 1.6.2, Willich, Germany) and printed with a Wanhao Duplicator 4 (Jinhua, China), which has a layer resolution of 100 μm, according to the provided user manual specifications. The maximum printing size was 25 × 25 × 3 mm^3^ (length, width, and height, respectively) with a layer height of 0.3 mm. The minimum resolution of the design and dimensions of the final pieces are depicted in Figure S1, Supplementary Materials. The AF/PLA filaments with different agave fiber weight percentages (0, 3, 5, 10 wt%) were printed using a nozzle temperature of 190 °C, a bed temperature of 70 °C and filled using a crosshatch pattern. The infill was set to 100%, with a crosshatch angle of −45°/45° and 0°/90°. The printing speed was 50 mm s^−1^.

### 2.6. Density and Porosity

Experimental density $\rho_{\exp},$ (g cm^−3^) calculations were performed for AF/PLA printed pieces of 2.5 × 2.5 × 0.3 cm^3^. The theoretical density was obtained from Equation (2).

(2) $${Ρ}_{\mathrm{th}}=\frac{1\;}{\frac{x_{\mathrm{PLA}}}{\rho_{\mathrm{PLA}}}+\frac{x_{\mathrm{AF}}}{\rho_{\mathrm{AF}}}}$$

where $\rho_{\mathrm{th}}$ is the theoretical density in g cm^−3^, $x_{\mathrm{PLA}},{\;x}_{\mathrm{AF}}$ are the weight fraction of PLA and AF, respectively, and $\rho_{\mathrm{PLA}},{\;\rho }_{\mathrm{AF}}$ are the experimental densities of PLA and AF, respectively, obtained via a 3P Instruments gas pycnometer Ultrapyc 1200e (Odelzhausen, Germany) with nitrogen in the chamber cell.

The porosity was calculated comparing the experimental and theoretical density as in [35], using Equation (3). All the experiments were performed using four samples for each composition.

(3) $$\mathrm{Porosity}\;\left(\%\right)=\frac{\rho_{\mathrm{th}}-\rho_{\exp}\;}{\rho_{\mathrm{th}}}$$

### 2.7. Water Absorption

The water absorption of AF/PLA printed composites was measured following the ASTM D570-03 standard at room temperature. Prior, AF/PLA printed pieces of 25 × 25 × 3 mm^3^ were dried in an oven at 60 °C and then weighed. Then, the samples were submerged in distilled water and removed after 2, 4, 6, and 24 h for 50 days, cleaned of excess water with a soft cotton cloth, and weighed for weight increment monitoring. All the experiments were performed using four samples for each composition.

### 2.8. Mechanical Properties

Tensile tests were carried out following the ASTM International D638-03 standard using type IV specimens. The testing was performed with an INSTRON 3345 (Norwood, MA, USA) with a 5 kN load cell at a crosshead speed of 5 mm/min, subjecting a total of 8 specimens to uniaxial tensile stress.

Flexural properties were obtained according to the ASTM International D790-03 standard. The three-point flexural testing was performed with the same machine used for tensile tests at a crosshead rate of 2 mm/min using 8 specimens of 70 × 12.7 × 3 mm^3^.

Impact strength tests were carried out in a Charpy impact tester (Tinius Olsen IT104 (Horsham, PA, USA)) using a pendulum of 242 g (1.22 J). Printed pieces were prepared according to the ASTM International D6110-04 standard. A manual sample notcher (Instron CEAST 6897 (Norwood, MA, USA)) was used to prepare the samples at least 24 h before testing. Each value represents the average of 8 notched samples. All samples were tested at room temperature (~25 °C). The load direction applied on the specimens with respect to the printing angle for the different mechanical tests is shown in Figure S2, Supplementary Materials.

### 2.9. Disintegration under Simulated Composting Conditions

Gravimetric weight loss of AF/PLA printed pieces was performed following the ISO standard 20200:2004. Initially, 25 × 25 × 3 mm^3^ pieces were oven-dried at 60 °C, until a constant weight was achieved. Then, the dried pieces were deposited under simulated composting conditions, with 50% organic matter, 58 °C and >40% of humidity for 49 days. Every 7 days, the composted specimens were withdrawn, cleaned, dried at 60 °C and weighed again to study the material weight lost. All experiments were performed by triplicate using 8 specimens for each composition.

### 2.10. Morphological Characterization

Micrographs were obtained with a field emission scanning electron microscope (FE-SEM) (Tescan MIRA3 LMU (Brno, Czech Republic)) using a laser beam of 10 kV accelerating voltage. Prior, the samples were immersed in liquid nitrogen, fractured, and then coated with a thin conductive layer of Au under vacuum using an SPI Module Sputter Coater (West Chester, PA, USA) for 60 s.

### 2.11. Statistical Analysis

The mechanical test data were subjected to an analysis of variance (ANOVA) to determine if the differences were statistically significant (*p* ≤ 0.05) between the evaluated factors and their levels. For each test, 8 repetitions were performed (8 specimens were fractured), and the two end values were discarded.

A multifactorial design with two factors—fiber content and printing angle—was designed with four and two levels, respectively. To analyze the behavior of the effects of fiber content, printed angle, and their interactions, the mean comparison was analyzed through the LSD test. The analyses were performed using Statgraphics Centurion XV (The Plains, VA, USA) Version 15.2.06 [36].

## 3. Results and Discussion

### 3.1. SEM Micrographs of Agave Fibers and AF/PLA Filaments

**Figure 1:**
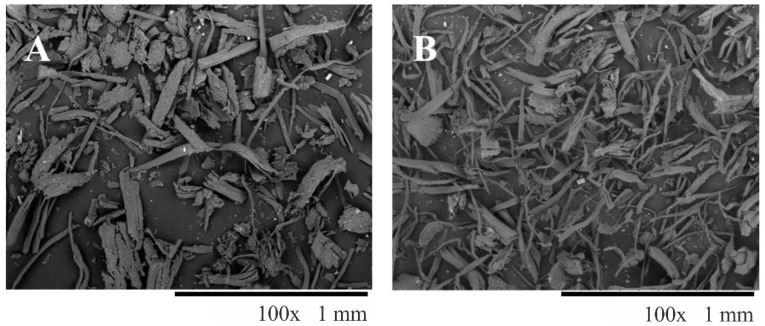
SEM micrographs of agave fibers (AF) used for biocomposite preparation (**A**) after sieving, and (**B**) after high shear mixing process (100× magnification).

**Figure 2:**
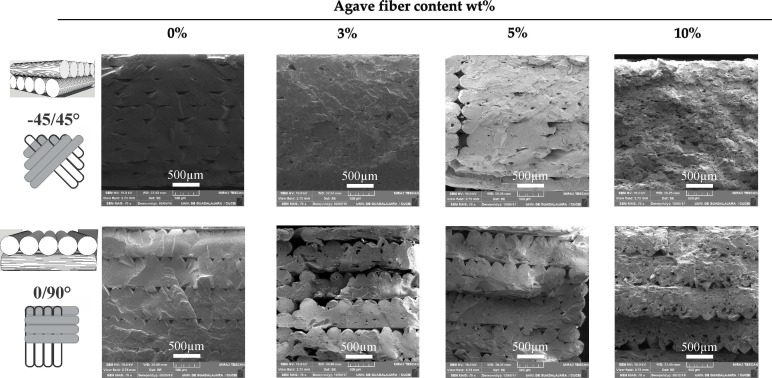
SEM micrographs of agave fibers/PLA biocomposites with different agave fiber content (0, 3, 5 and 10 wt%) and angle deposition (−45°/45° and 0°/90°) (70× magnification).

The surface morphology of AF is detailed in Figure 1. AF were used without any chemical treatment; therefore, impurities and debris can be observed in Figure 1A. Before filament preparation, the diameter and length of the AF were measured from SEM micrographs. Properties such as fiber length and fiber orientation can alter the final mechanical properties of a given composite [29]. Damaged fibers by compression-molding show a steady reduction in fiber length, thus compromising its filler ability [37]. The measured area was converted to an equivalent diameter of a circle to obtain the fiber diameter. Clean AF (400 fibers counted, Figure 1A) showed an equivalent diameter of 46.6 ± 20.9 μm and a length of 246 ± 92 µm (aspect ratio, L/D, of 5.2). After the sieving process (Figure 1B), AF had a diameter of 37.7 ± 16.6 µm and a length of 255 ± 108 µm (capillary L/D ratio of 6.7). Sieved AF showed a difference in L/D aspect ratio compared to clean AF due to a decrease in their diameter. After the extrusion process, the AF/PLA filaments with 10 wt% agave content were frozen with liquid nitrogen, and then, the filament diameter (100 fibers counted) was measured directly from cryofracture SEM micrographs (Figure 2). The obtained diameter was 36.6 ± 16 µm. However, the fiber length in the final printed piece could not be measured. Nonetheless, damage to some extent in the length is expected [37].

### 3.2. Thermal Properties and Crystallinity of Agave Fibers/PLA Filaments

Values for glass transition ($T_{g}$), crystallization ($T_{c}$) and melting temperatures ($T_{m}$) were obtained from the DSC curves for neat PLA and AF/PLA filaments (Table 1). While $T_{g}$ and $T_{c}$ values diminished with the addition of AF, $T_{m}$ values of filaments showed a minor increase with respect to PLA. The 0–3 wt% and 5–10 wt% AF/PLA filaments showed a 5% and 11% reduction in $T_{c}$ values, respectively, in comparison to neat PLA. $T_{g}$ values for neat PLA and 0 wt% AF/PLA filaments were similar (58.9 °C and 59.1 °C, respectively), whereas $T_{g}$ values for 3–10 wt% AF/PLA filaments showed no significant differences (57.9 °C, 57.7 °C, and 57.2 °C for 3 wt%, 5 wt%, and 10 wt% AF/PLA composites, respectively). The DSC thermal transitions for neat PLA and all AF/PLA filaments are detailed in Figure S3, Supplementary Materials.

The crystallinity values of PLA and AF/PLA filaments were obtained from ${\Delta H}_{c}$ and ${\Delta H}_{f}$ values. After the extrusion process, the crystallinity value of 0 wt% AF/PLA filaments increased from 11.6% to 23.7% (Table 1), which can be attributed to the cooling rate of the filaments after the extrusion stage [38]. The low crystallinity value observed for neat PLA pieces is explained by PLA stoichiometry (L and D isomers) and the slow crystallization rate of PLA [39]. High D content gives amorphous extruded materials. In this work, the low crystallinity value of PLA 3251 is due to the presence of 1.4% of the D-isomer.

The crystallinity values of AF/PLA filaments were similar for biocomposites of between 0–3 wt% and 5–10 wt% AF content. However, the values increased up to 50% and up to 71% for 0–3 wt% and 5–10 wt% AF content, respectively, compared to neat PLA. This effect can be attributed to the nucleation effect caused by the presence of AF [40]. Similarly, Teixeira et al. found that after adding 10 wt% of thermoplastic bagasse to PLA biocomposites, their crystallinity increased from 1.6 to 17.5%, due to the presence of cellulosic fibers from cassava bagasse [41].

### 3.3. FDM Deposition Angle and Agave Fiber Content

In FDM, variables such as printing speed, printing temperature, layer thickness, and pattern, among others, directly influence the quality and mechanical properties of the final fabricated pieces [2,42]. In the present work, AF/PLA biocomposites were printed horizontally since higher mechanical properties have been reported for printed pieces in this particular direction with the presence of a contour layer in all cases [2,43,44].

Figure 2 shows the SEM micrographs of the printed pieces at different printing angles and AF content. In pieces printed at 0°/90°, the fracture has a characteristic transverse shape, parallel to the printing threads. The layer thickness value obtained at 0°/90° was close to the specified printing value (0.3 mm) and diminished to 0.26 mm when the AF content increased to 10 wt%. The thickness at −45°/45° had an average of 0.27 mm (Table S1, Supplementary Materials). However, the thicknesses of the AF/PLA biocomposites printed at −45°/45° were measured with a higher error due to the fusion of the adjacent layers. In addition, a change in morphology was observed in specimens printed using both configurations −45°/45° and 0°/90°, which is directly proportional to the increase in fiber content.

### 3.4. Density and Porosity of Agave-Fiber/PLA Printed Biocomposites

Printed biocomposites with 0 wt% AF/PLA showed 7% and 7.5% porosity attributed to the interstices formed between the printing threads in the FDM process (Table 2). This issue is intrinsically related to the FDM technique, and the presence of voids is expectedly higher than the obtained with pieces fabricated with other processing methods, such as injection molding [24]. In addition to the process parameters, variables such as material composition, printing angle, and printing speed greatly influence the final porosity of the biocomposites. For example, the porosity values of 3D-printed carbon fibers in ABS composites were found to be highly dependent on the printing angle, and specimens printed at 0°/90° showed much more porosity compared to specimens printed at −45°/45° [45]. In this work, the highest AF content tested for AF/PLA biocomposites printed at −45°/45° caused an increase in porosity by 2.8-fold (7.0% ± 1.9 to 20.1% ± 0.5 for biocomposites with 0 wt% and 10 wt% AF, respectively) and 2.9-fold for biocomposites printed at 0°/90° (7.5% ± 0.8 to 21.9% ± 1.3 for biocomposites with 0 wt% and 10 wt% AF, respectively). Thus, higher concentrations of AF strongly influenced the porosity of the final biocomposites rather than the raster angle. The presence of natural fibers on polymeric composites is known to induce the formation of porous structures due to the chemical incompatibility between the used materials, also known as interfacial voids [29,46]. This is expected to negatively affect the mechanical properties, as the filler capacity is reduced [47].

Similarly, apparent experimental densities of biocomposites decreased with increased AF percentages for biocomposites printed at −45°/45° (1.159 g cm^−3^ ± 0.023 to 0.999 g cm^−3^ ± 0.006 for specimens with 0 wt% and 10 wt% AF, respectively) and 0°/90° (1.153 g cm^−3^ ± 0.009 to 0.976 g cm^−3^ ± 0.017 for specimens with 0 wt% and 10 wt% AF, respectively) (Table 2). The presence of voids and fibers can be observed in Figures S4 and S5, Supplementary Materials. As the mass fraction of the AF is lower compared to the mass fraction of PLA, the global contribution of fibers in the density of the biocomposites is not significant; therefore, the density of the composite is expected to be similar to that of PLA (1.246 and 1.278 g cm^−3^, respectively). However, the density value of AF is still significantly lower than that of conventional reinforcements, such as fiberglass (density value of 2.55 g cm^−3^) [48]. Pérez-Fonseca et al. produced biocomposites with AF and pine fiber with polypropylene as the matrix, reporting that as the natural fiber content increased, the density of the fabricated composite also decreased [49].

### 3.5. Water Absorption

**Figure 3:**
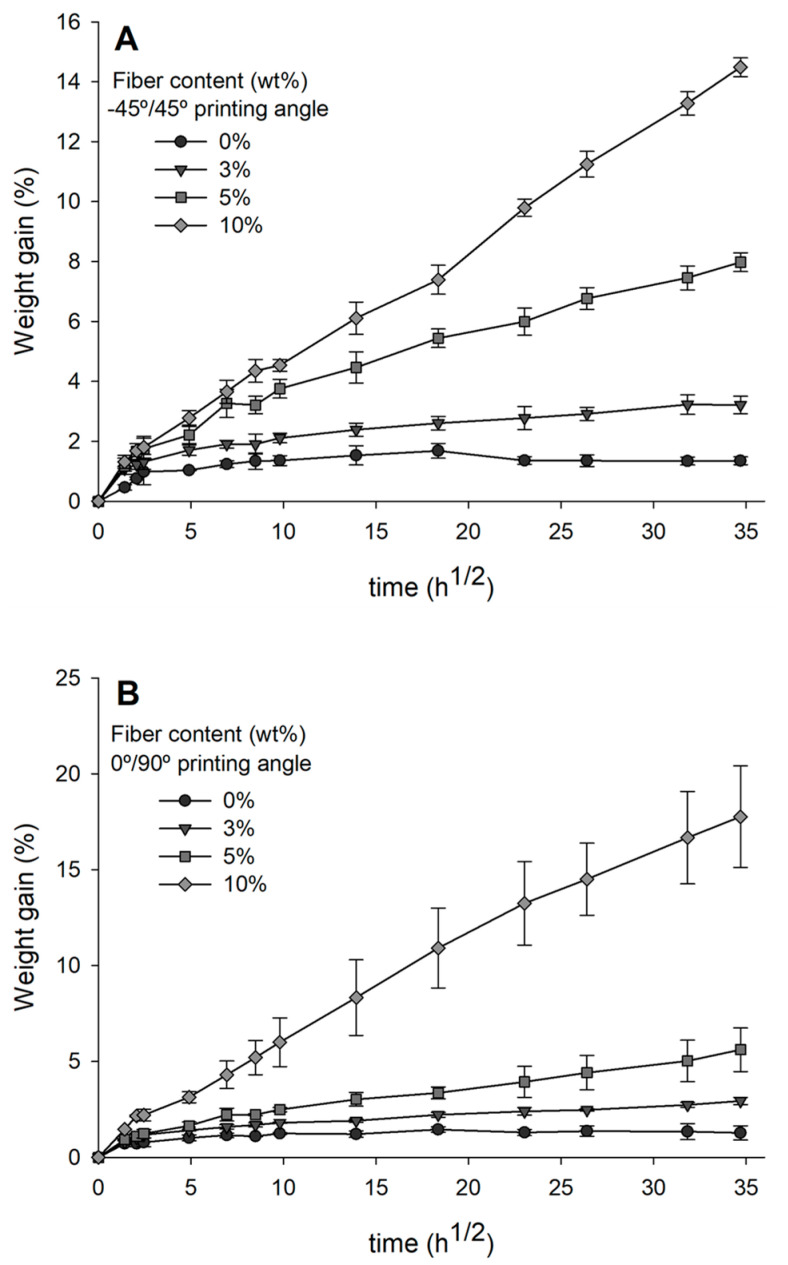
Water absorption of agave fibers/PLA printed biocomposites at different agave fiber percentages (0, 3, 5, and 10 wt%) and (**A**) printing angle of −45°/45°, and (**B**) printing angle of 0°/90°.

Water absorption is a process where multiple factors intervene, such as the homogeneity of the sample, the presence of fibers, and the presence of voids within the pieces [50]. Specimens fabricated from PLA can be susceptible to increased water absorption, as the polar bonds of PLA may decompose in the presence of water, thus changing the mechanical properties of the final piece [51]. Figure 3 shows the gained weight versus t12 curves of FDM printed AF/PLA biocomposites at different percentages and two printing angles. A rapid increase in water absorption is observed until 150 h, followed by a saturation after 1225 h, except for 5 and 10 wt% AF/PLA biocomposites. A similar behavior is also observed for biocomposites of kenaf bast fiber and PLA [52]. A strong correlation of the printing angle with water absorption is observed for biocomposites with 3% and 5% AF. In these cases, the water absorption was superior for pieces printed at −45°/45°. In contrast, for 10 wt% AF/PLA biocomposites printed at 0°/90°, the water uptake was higher than that for biocomposites printed at −45°/45°. Natural fibers can form gaps, voids, and cracks because of fiber swelling. Thus, water is expected to flow through the gaps via capillary action [52]. This could explain the increased values of water uptake showed for specimens with 10 wt% AF.

The ‒OH groups of AF should increase the hydrophilicity of the final material, as these groups attract water molecules via hydrogen and van der Waals bonds [53]. This effect was observed with sisal fiber/PLA biocomposites, which showed a 3.7-fold increase in water absorption compared to neat PLA, due to the presence of the highly hydrophilic sisal fibers [54]. Similarly, Le Duigou et al. demonstrated that increasing porous structures and voids have a more pronounced effect on water absorption than the sole presence of natural fibers [55]. However, we observed that water uptake values did not correlate with increasing AF content. Instead, the tendency matched the amount of porosity present in the material, which in turn correlated with the printing angle.

### 3.6. Tensile Properties

**Figure 4:**
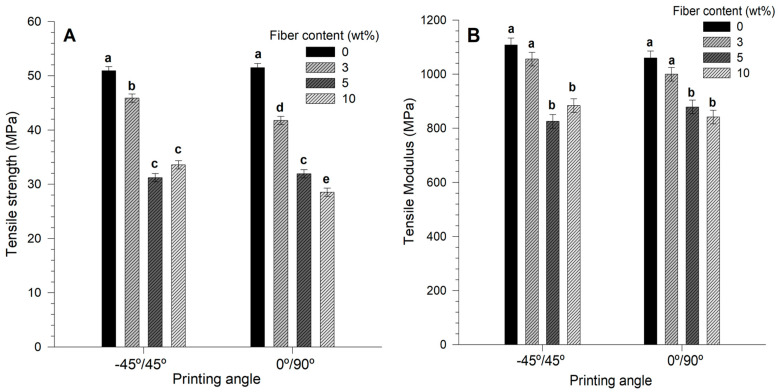
(**A**) Tensile strength and (**B**) tensile modulus of printed agave fibers/PLA biocomposites. The letters a–e on top of the bars indicates significant differences (*p* < 0.05).

Increasing fiber content caused a decrease in the tensile properties of 3D-printed AF/PLA biocomposites, as shown in Figure 4. FDM printed biocomposites with 0 wt% AF had similar tensile strength and modulus values for both printed angles (~51 MPa). The lowest tensile strength value was recorded for biocomposites with 5 wt% AF at −45°/45° (31 MPa), and biocomposites with 10 wt% AF/PLA at 0°/90° (28 MPa), which represents a 1.8-fold decrease in the tensile strength compared to neat PLA pieces.

It should be noted that the tensile strength values obtained for 10 wt% AF/PLA printed biocomposites (28 MPa) are higher compared to the values of biocomposites processed with different techniques, i.e., compression molding of AF/PHB (14 MPa) and AF/LMDPE biocomposites (13 MPa) [28,56]. In contrast, compared to other natural fiber fillers for FDM processes, AF are better filler options than wood flour/PLA composites [20], wood/PLA composites [21], and cork/PLA composites [23]. Nevertheless, the mechanical behavior of AF/PLA is lower compared to reinforced sugarcane bagasse fiber/PLA biocomposites (tensile strength of 55 MPa with 6 wt% fiber content) [57].

Similarly, the tensile modulus decreased with the increasing AF content of printed specimens at 0°/90°. At 10 wt% AF, the tensile value was 1.25-fold lower (880 MPa) compared to that of 0 wt% AF. For specimens printed at −45°/45°, the tensile modulus values decreased from 0 to 5 wt%, reaching a minimum at 5 wt% AF (825 MPa). Finally, at 10 wt% AF content, the tensile modulus value increased by 1.1-fold (900 MPa) in comparison to specimens with 5 wt% AF.

The addition of natural fibers is expected to increase the stiffness (tensile modulus) of a given composite as observed by compression and injection techniques [40,56]. Tensile modulus values for PLA are around 1700 MPa processed by rotational molding [29,38], and 2300 MPa for injection [40,58]. By FDM, a lower tensile modulus value was obtained for neat PLA (1100 mPa), probably due to a higher porosity and a different crystallinity value of the final material caused by the FDM process [59].

### 3.7. Flexural Properties

**Figure 5:**
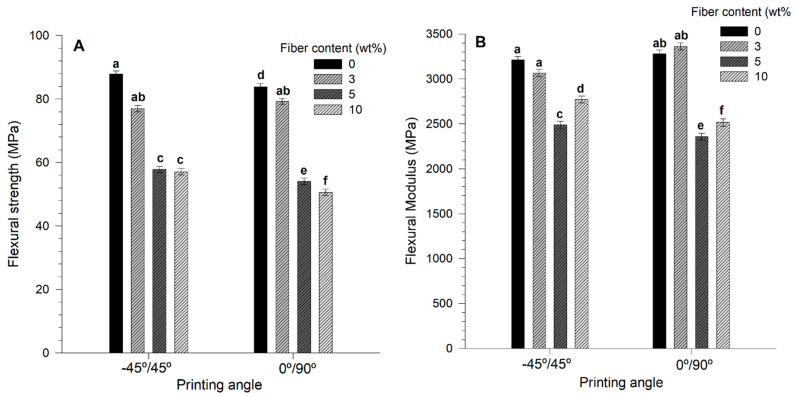
(**A**) Flexural strength and (**B**) flexural modulus of printed agave fibers/PLA biocomposites. The letters a–f on top of the bars indicates significant differences (*p* < 0.05).

Figure 5 shows the obtained flexural properties for AF/PLA biocomposites at different raster angles. In general, the addition of fibers had a detrimental effect on the flexural strength values, regardless of the printing angle. The maximum flexural strength value was observed when no AF were used (0 wt% AF biocomposites, 87 and 82 MPa for −45°/45° and 0°/90°, respectively), followed by biocomposites with 3 wt% AF for both printing angles (79 and 77 MPa for −45°/45° and 0°/90°, respectively). The flexural strength values of biocomposites printed at −45°/45° with 5 wt% and 10 wt% AF/PLA decreased by 28 MPa compared to biocomposites with 0 wt% AF (87 MPa compared to 59 MPa). Biocomposites printed at 0°/90° with 10 wt% AF showed the lowest flexural strength value recorded (51 MPa), which represents a 1.7-fold decrease compared to biocomposites printed at the same angle and no fibers present. Similar results have been observed for 3D-printed sugarcane bagasse fiber/PLA composites [57] and rice straw/ABS composites [42], where flexural properties decrease as fiber concentration increases.

Similarly, the flexural modulus diminished with the addition of fibers, and the recorded values were similar for both raster angles. At −45°/45°, 0 wt%, and 3 wt% AF/PLA biocomposites showed flexural modulus values around 3100 MPa, while the minimum flexural modulus value of 2500 MPa was obtained with 5% AF pieces. However, 3 wt% AF/PLA biocomposites printed at 0°/90° showed an increase by 94 MPa in the flexural modulus compared with biocomposites with 0 wt% AF/PLA (3374 compared to 3280 MPa). The lowest flexural modulus value (2350 MPa) was observed for specimens printed with 5 wt% AF and 0°/90°.

Although 0 wt% AF/PLA biocomposites showed flexural strength values slightly lower than those reported in the literature for different processing techniques, the obtained flexural modulus values were higher than the tendency observed for PLA pieces produced via other techniques, such as injection, rotational molding, and compression (2300, 3700 and 3100 MPa, respectively) [29,38,40].

The flexural properties observed for the biocomposites printed in this work showed no dependence with the printing angle. However, other authors have observed a correlation between printing angle and tensile and flexural strength, with a general decrease in the values from −45°/45° to 0°/90°. For pieces printed at 0°/90°, only the layers printed at 0° with respect to the axis are those that support the tensile test, while the ones at 90° had a minor effect opposing the pulling forces [59]. This behavior could be because for both printing angles (−45°/45° and 0°/90°), a network of wires is formed perpendicularly to each angle, which achieves a similar load distribution [60]. When pieces were built at a single angle through all the layers, the load was distributed differently. Cole et al. (2016b) produced pieces by varying the printing angle and found that pieces printed at 0° showed greater tensile strength after those manufactured at 45° and 0°/90°, which may be because, at 0°, the printing threads are oriented parallel to the applied load [44].

### 3.8. Impact Properties

**Figure 6:**
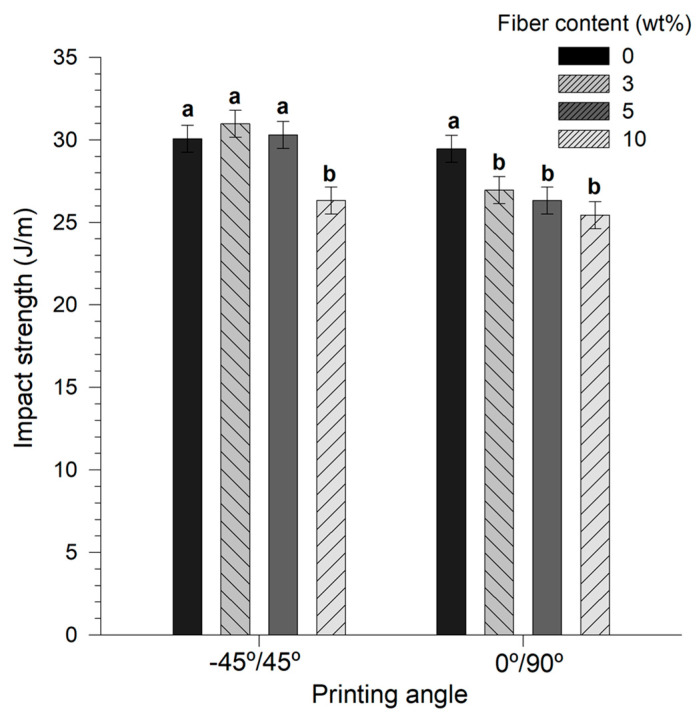
Charpy impact strength of printed agave fibers/PLA biocomposites. The letters a–b on top of the bars indicates significant differences (*p* < 0.05).

In Figure 6, the impact strength for AF/PLA biocomposites as a function of AF content and raster angle is depicted, where a positive correlation is observed for a raster angle of −45°/45°. For biocomposites printed at −45°/45°, the impact strength value increased by 3% for 3 wt% AF/PLA compared to neat PLA. Increasing the AF content to 10 wt% decreased the impact strength by 1.1-fold with 27 J m^−1^. In contrast, the impact resistance values for specimens printed at 0°/90° decreased steadily with increased AF content, reaching a minimum value of 26 J m^−1^, with no significant difference compared to the 10 wt% AF/PLA printed at −45°/45°.

The properties of a biocomposite rely on the chemical and physical nature of matrix and fibers. Increasing the fiber content in the AF/PLA biocomposites obtained in this work decreased the impact resistance of the material. This is explained by a low fiber–matrix compatibility causing defects and stress regions, which can be enhanced by the use of short fibers [24,29].

In our case, this phenomenon was more pronounced with pieces printed at 0°/90°, with half of the printing threads in a parallel position to the crack propagation, which absorbs less energy [61].

### 3.9. Disintegration under Simulated Composting Conditions

**Figure 7:**
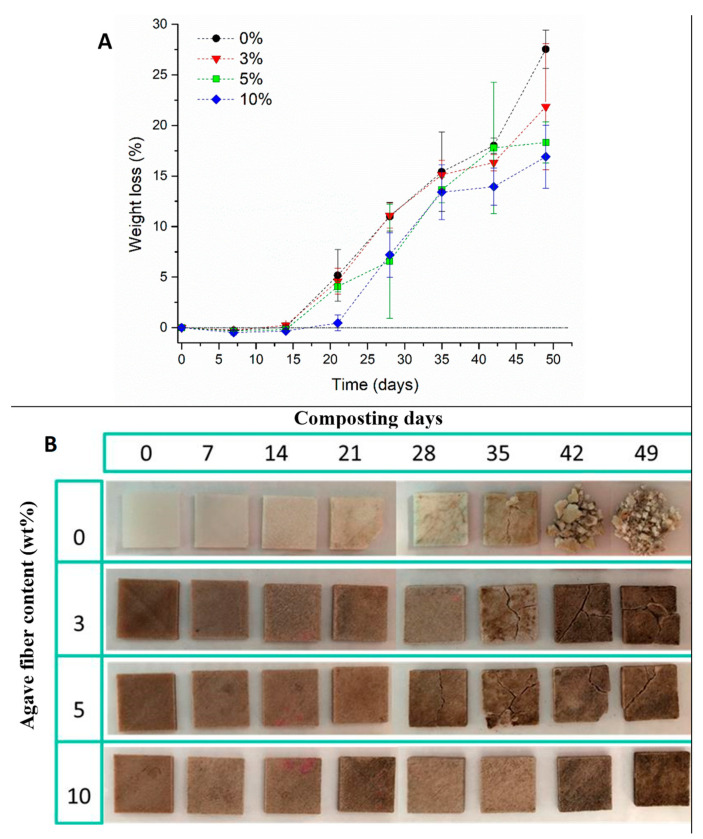
(**A**) Percentage weight losses of agave fiber/PLA printed biocomposites under simulated composting conditions and (**B**) photographs of agave fiber/PLA pieces after being composted.

The simulated composted conditions for specimens printed at −45°/45° showed no changes in weight during the initial 15 days of the experiment (Figure 7), which could be attributed to microbial lag and the adaptation to the hydrolysis of PLA chains, while the small changes observed are attributed to weighting errors. After 21 days of composting, losses of 5% in weight for 0, 3, and 5 wt% AF/PLA were observed, while 10 wt% AF/PLA pieces showed a lag phase. These changes can be explained by the generation of small oligomers and PLA monomers, which can be metabolized by microorganisms present in the compost material. After 35 days, the weight loss was around 15% for all the printed biocomposites, with no statistical differences. After 49 days of composting, 0 wt% AF/PLA biocomposites showed the maximum weight loss (~27% weight loss), followed by biocomposites with 5 wt% and 10 wt% AF (~17% weight loss). In contrast, printed biocomposites with 3 wt% AF had an average of 22% weight loss; however, the standard deviation observed was 6%. The total weight loss after 50 days of 10 wt% AF/PLA specimens was 1.6-fold less than the weight loss observed for neat PLA specimens. These results can be attributed to an increased crystallinity observed with increased AF contents, as crystalline regions are less susceptible to degradation than amorphous regions [62,63]. Similarly, Dong et al. obtained zero net weight losses with coir fiber/PLA from 6 to 12 days, with an 18.1% reduction in weight loss after 18 days [64]. In this case, the presence of coir fibers increased the weight loss up to 34.9%, due to the high hydrophilicity of the final composites. Under environmental conditions, the decomposition of PLA to carbon dioxide, water, and methane can take from several months up to 2 years [50]. On the other hand, the fact that the composites based on AF presented a preserved structure after the composting test could be associated with an increased PLA crystallinity and stiffness by thermal annealing at the test conditions (58 °C) [40]. A brittle PLA could explain the high fragmentation of samples; in this case, the AF helped to keep the physical structure of the composite samples, without compromising their biodegradability.

Additional optical micrographs of the disintegration of the pieces are shown in Figure S6, Supplementary Materials.

## 4. Conclusions

In this work, AF were extruded with PLA and the filaments were employed to prepare AF/PLA biocomposites by FDM. After the extrusion process, the AF suffered mechanical damage as the fiber diameter was reduced. The results herein indicated that the fiber content, rather than the raster angle, strongly influenced the crystallinity, porosity, flexural and strength properties of the final biocomposites. In contrast, the raster angle influenced the morphology and impact resistance of the printed biocomposites. This is highly significant for future fabrications with FDM, as the choice of the natural fiber, with inherent features, as well as the thermoplastic polymer, ultimately affects the characteristics and applicability of the final piece. The fabricated AF/PLA printed biocomposites in this study have proven to be a low-cost and compostable biocomposite, with lower disintegration rate and density (lightweight). This feature can be useful for the fabrication of molds and 3D printing support materials based on AF, such as toys, which could be easily discarded and composted after use.

Although further research is needed, AF can be employed as a component for 3D printing, and the filling of PLA composites with natural fibers is an attractive, compostable, and environmentally friendly alternative over traditional filler materials.

## Figures and Tables

**Table 1:** Thermal properties and crystallinity values of the filaments.

Filament	Agave Fiber Content (wt%)	$T_{g}$ (°C)	$T_{cc}$ (°C)	$T_{m}$ (°C)	$\Delta H_{cc}\;(J\;g{-}^{1})\;*$	$\Delta H_{m}(J\;g{-}^{1})\;*$	Crystallinity (%)
Neat PLA		58.9	95.9	168.0	33.7	44.5	11.6
Agave fiber/PLA filament	0	59.1	91.2	168.9	25.8	48.0	23.7
	3	57.9	91.8	169.3	25.3	46.1	22.9
	5	57.7	85.2	168.0	12.6	49.7	41.7
	10	57.2	84.7	169.0	11.4	48.5	44.1

* The enthalpy values were normalized for the polymer content.

**Table 2:** Effect of agave fiber content on apparent density and porosity of the agave fibers/PLA printed pieces.

Agave Fiber Content (wt%)	Theoretical Density (g cm^−3^)	−45°/45°		0°/90°	
		Experimental Density (g cm^−3^)	Porosity (%)	Experimental Density (g cm^−3^)	Porosity (%)
0	1.247	1.159 ± 0.023	7.0 ± 1.9	1.153 ± 0.009	7.5 ± 0.8
3	1.248	1.104 ± 0.024	11.5 ± 1.9	1.135 ± 0.015	9.0 ± 1.2
5	1.249	0.989 ± 0.016	20.8 ± 1.3	1.042 ± 0.007	16.6 ± 0.6
10	1.250	0.999 ± 0.006	20.1 ± 0.5	0.976 ± 0.017	21.9 ± 1.3

## Data Availability

The data presented in this study are available on request from the corresponding author.

## Supplementary Materials

The following are available online at https://www.mdpi.com/article/10.3390/ma14113111/s1, Figure S1: Computer-aided designs (CAD) and their dimensions following the ASTM standards for A) water absorption and biodegradation tests, (B) tensile tests, (C) flexural tests, and D) impact tests; Figure S2: Load direction applied on the specimens with respect to the printing angle for the different mechanical tests. (A) tensile, (B) flexural, and (C) impact; Figure S3: Differential scanning calorimetry (DSC) thermograms of first heating for raw PLA and agave fiber filaments; Figure S4: Scanning electron microscope (SEM) micrographs of the printed pieces showing the presence of voids and fibers; Figure S5: Scanning electron microscope (SEM) micrographs of agave fiber/PLA biocomposites printed at −45°/45° showing agave fiber details. (A) 0 wt% agave fiber/PLA biocomposites, (B) 3 wt% agave fiber/PLA biocomposites, (C) 5 wt% agave fiber/PLA biocomposites, and (D) 10 wt% agave fiber/PLA biocomposites; Figure S6: Optical micrographs of agave fiber/PLA biocomposite pieces under simulated composting conditions obtained after being weighed. Table S1: Formulation of biocomposites; Table S2: Layer thickness in cm for 3D-printed agave fiber/PLA biocomposites at −45°/45° and 0°/90° measured from SEM micrographs.
