# Supplementary material for: Mechanical and Physicochemical Properties of 3D-Printed Agave Fibers/Poly(lactic) Acid Biocomposites
Source: Materials (Basel). 2021 Jun 5;14(11):3111. doi: 10.3390/ma14113111 (PMC8201365; doi:10.3390/ma14113111)
Supplement: Supplementary file 1 [file materials-14-03111-s001.zip › materials-1220046-supplementary.pdf]

materials

Supplementary Materials

Mechanical and Physicochemical Properties of 3D-Printed Agave Fibers/Poly(lactic) Acid Biocomposites

Valeria Figueroa-Velarde, Tania Diaz-Vidal, Erick Omar Cisneros-López, Jorge Ramón Robledo-Ortiz, Edgar J. López-Naranjo, Pedro Ortega-Gudiño and Luis Carlos Rosales-Rivera *

University Center of Exact Sciences and Engineering (CUCEI), University of Guadalajara, 44430 Guadalajara, Mexico; valeria.fvelarde@alumnos.udg.mx (V.F.-V.); taniadzv@gmail.com (T.D.-V.); erick.cisneros@academicos.udg.mx (E.O.C.-L.); jorge.robledo@academicos.udg.mx (J.R.R.-O.); edgar.lopezn@academicos.udg.mx (E.J.L.-N.); pedro.ogudino@academicos.udg.mx (P.O.-G.)

* Correspondence: carlos.rosales@academicos.udg.mx (L.C.R.-R.); Tel.: +52 1 3313 7859 00 ext. 27590; Fax: +52 1 3313 7859 00 ext. 27590

Table S1. Formulation of biocomposites.

Agave Fiber Content (%)	PLA (g)	Agave Fiber (g)	Total Weight (g)
0	1000	0	1000
3	970	30	1000
5	950	50	1000
10	900	100	1000

Table S2. Layer thickness in cm for 3D-printed agave fiber/PLA biocomposites at −45°/45° and 0°/90° measured from SEM micrographs.

Agave Fiber Content (wt%)	−45°/45° (cm)	0°/90° (cm)
0	0.272 ± 0.018	0.296 ± 0.019
3	0.270 ± 0.011	0.281 ± 0.015
5	0.267 ± 0.024	0.293 ± 0.030
10	0.253 ± 0.014	0.271 ± 0.031

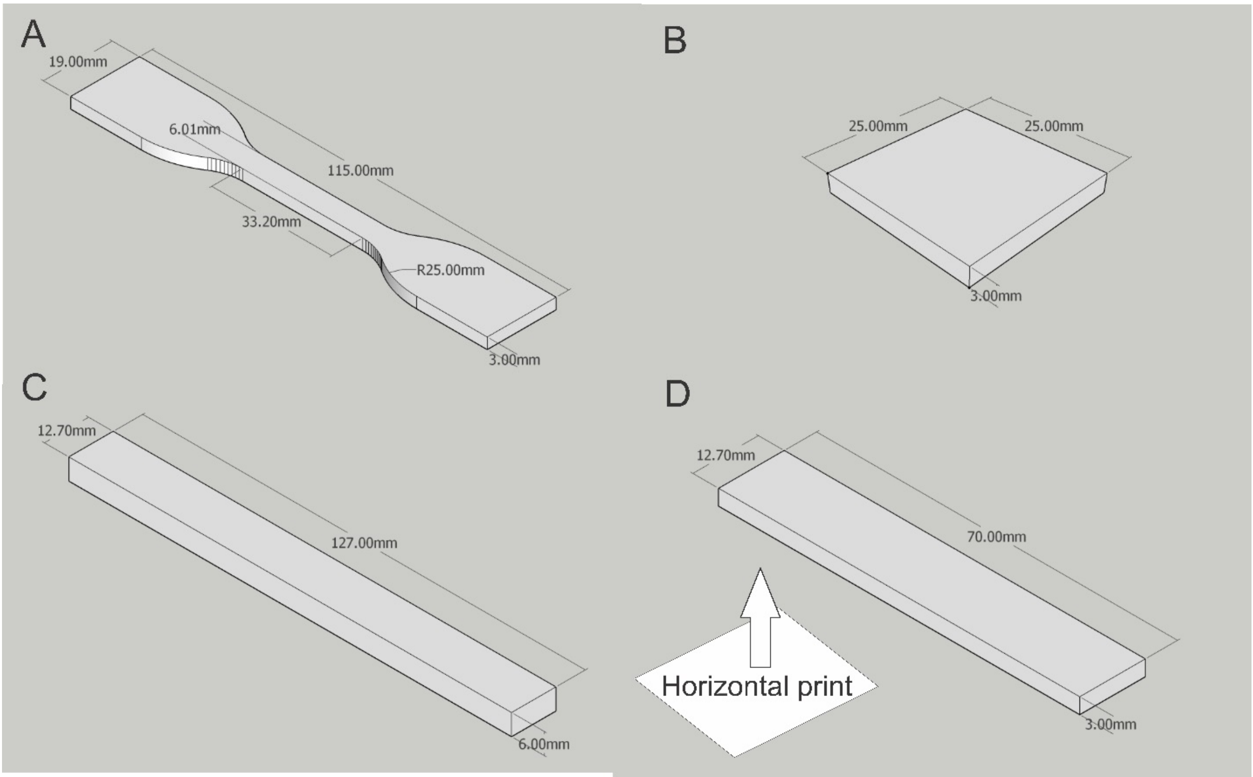

Figure S1. Computer-aided designs (CAD) and their dimensions following the ASTM standards for (**A**) water absorption and biodegradation tests, (**B**) tensile tests, (**C**) flexural tests, and (**D**) impact tests.

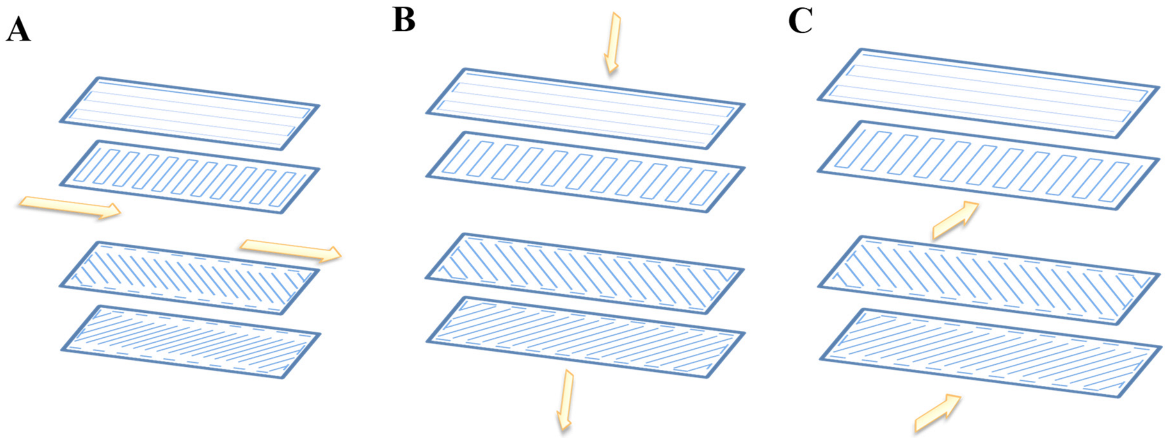

Figure S2. Load direction applied on the specimens with respect to the printing angle for the different mechanical tests. (**A**) tensile, (**B**) flexural, and (**C**) impact. The two upper sheets represent the printed parts at 0°/90°, while the two lower sheets represent the printed parts at −45°/45°.

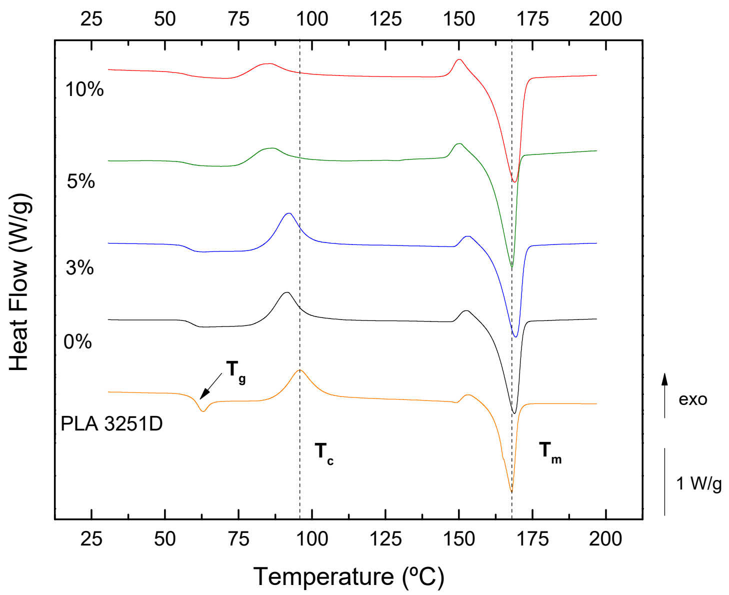

Figure S3. Differential scanning calorimetry (DSC) thermograms of first heating for raw PLA and agave fiber filaments.

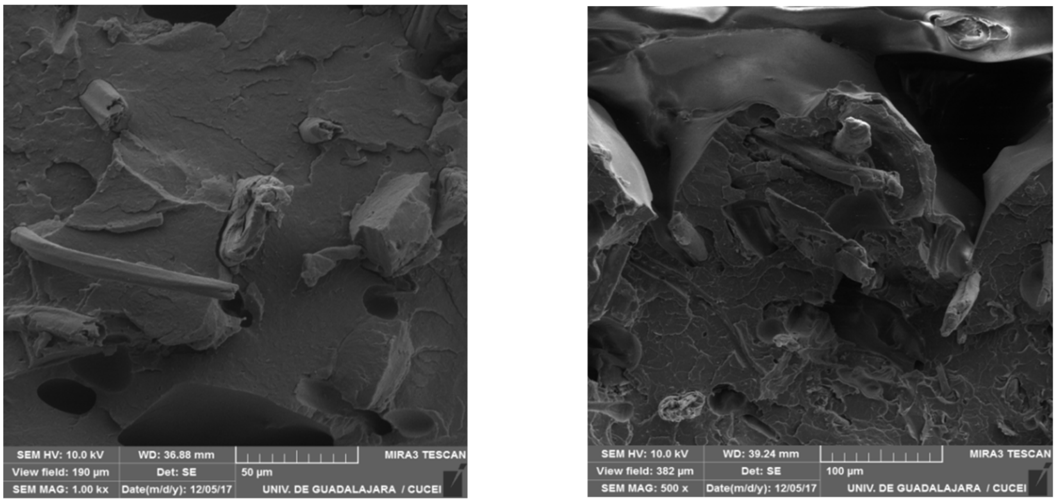

Figure S4. Scanning electron microscope (SEM) micrographs of the printed pieces showing the presence of voids and fibers.

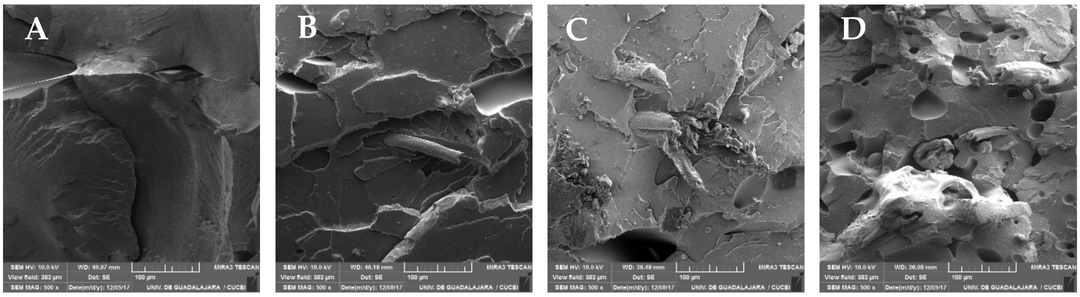

Figure S5. Scanning electron microscope (SEM) micrographs of agave fiber/PLA biocomposites printed at −45°/45° showing agave fiber details. (**A**) 0 wt% agave fiber/PLA biocomposites, (**B**) 3 wt% agave fiber/PLA biocomposites, (**C**) 5 wt% agave fiber/PLA biocomposites, and (**D**) 10 wt% agave fiber/PLA biocomposites (500× magnifications).

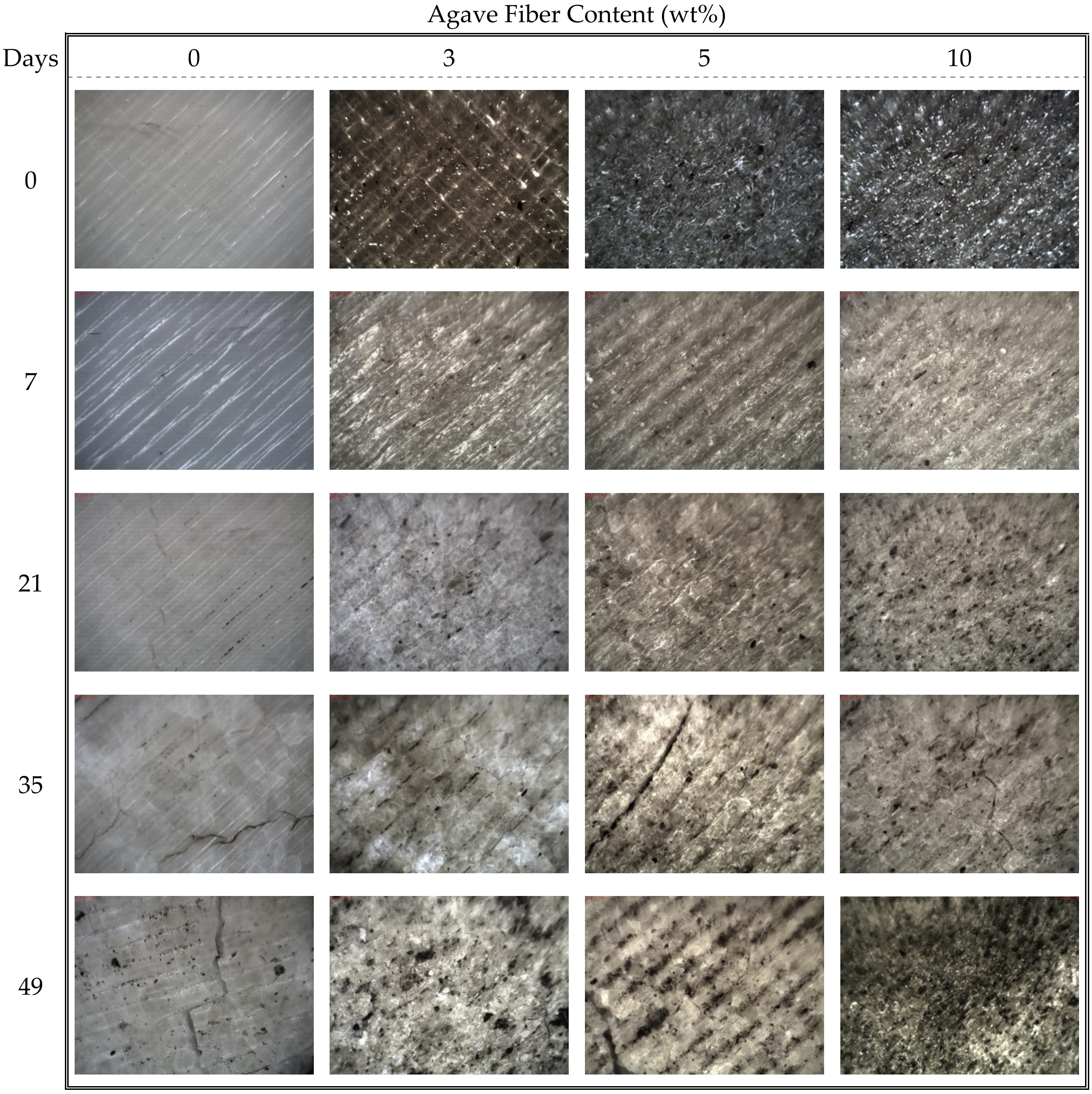

Figure S6. Optical micrographs of agave fiber/PLA biocomposite pieces under simulated composting conditions obtained after being weighed.
